# Unexpected Mechanism of Biodegradation and Defluorination of 2,2-Difluoro-1,3-Benzodioxole by Pseudomonas putida F1

**DOI:** 10.1128/mBio.03001-21

**Published:** 2021-11-16

**Authors:** Madison D. Bygd, Kelly G. Aukema, Jack E. Richman, Lawrence P. Wackett

**Affiliations:** a Microbial Engineering, University of Minnesota, St. Paul, Minnesota, USA; b Biochemistry, Molecular Biology and Biophysics, University of Minnesota, St. Paul, Minnesota, USA; c BioTechnology Institute, University of Minnesota, St. Paul, Minnesota, USA; University of Washington

**Keywords:** organofluorine, PFAS, defluorination, *Pseudomonas putida* F1, bacteria, dioxygenase, rapid rate, pyrogallol, fluoride, pesticides, oxygenase

## Abstract

Perfluorinated carbon atoms in a diether linkage are common in commercial anesthetics, drugs, fungicides, and insecticides. An important chemical group comprising perfluorodiethers is the 2,2-fluoro-1,3-benzodioxole (DFBD) moiety. The fluorine atoms stabilize the molecule by mitigating against metabolism by humans and microbes, as used in drugs and pesticides, respectively. Pseudomonas putida F1 catalyzed defluorination of DFBD at an initial rate of 2,100 nmol/h per mg cellular protein. This is orders of magnitude higher than previously reported microbial defluorination rates with multiply fluorinated carbon atoms. Defluorination rates declined after several hours, and the medium darkened. Significant defluorination activity was observed with cells grown on toluene but not l-arginine. Defluorination required only toluene dioxygenase. Pseudomonas and recombinant Escherichia coli cells expressing toluene dioxygenase oxidized DFBD to DFBD-4,5-dihydrodiol. The dihydrodiol could be oxidized to 4,5-dihydroxy-DFBD via the dihydrodiol dehydrogenase from P. putida F1. The dihydrodiol dehydrated with acid to yield a mixture of 4-hydroxy-DFBD and 5-hydroxy-DFBD. All those metabolites retained the difluoromethylene group; no fluoride or dark color was observed. The major route of DFBD-4,5-dihydrodiol decomposition produced fluoride and 1,2,3-trihydroxybenzene, or pyrogallol, and that was shown to be the source of the dark colors in the medium. A mechanism for DFBD-4,5-dihydrodiol transformation to two fluoride ions and pyrogallol is proposed. The Pseudomonas genome database and other databases revealed hundreds of bacteria with enzymes sharing high amino acid sequence identity to toluene dioxygenase from P. putida F1, suggesting the mechanism revealed here may apply to the defluorination of DFBD-containing compounds in the environment.

## INTRODUCTION

Fluorinated organic compounds are considered to be one of the major foci of biodegradation currently because of their prevalence, emerging concerns about health effects, and their prolonged lifetime in the environment (1). Naturally occurring compounds with carbon-fluorine bonds are relatively rare, and most contain a single fluorine substituent (2–4). Synthetic, commercially important fluorinated compounds typically contain multiple fluorine atoms, and that makes them less reactive to defluorination, both chemically and biologically (5, 6). In light of that, there have been a limited number of studies demonstrating biodegradation of multiply fluorinated compounds. Recently, perfluorinated compounds have been shown to be biodegradable (7, 8) but the genes and enzymes involved in their defluorination are not yet known.

**FIG 1:**
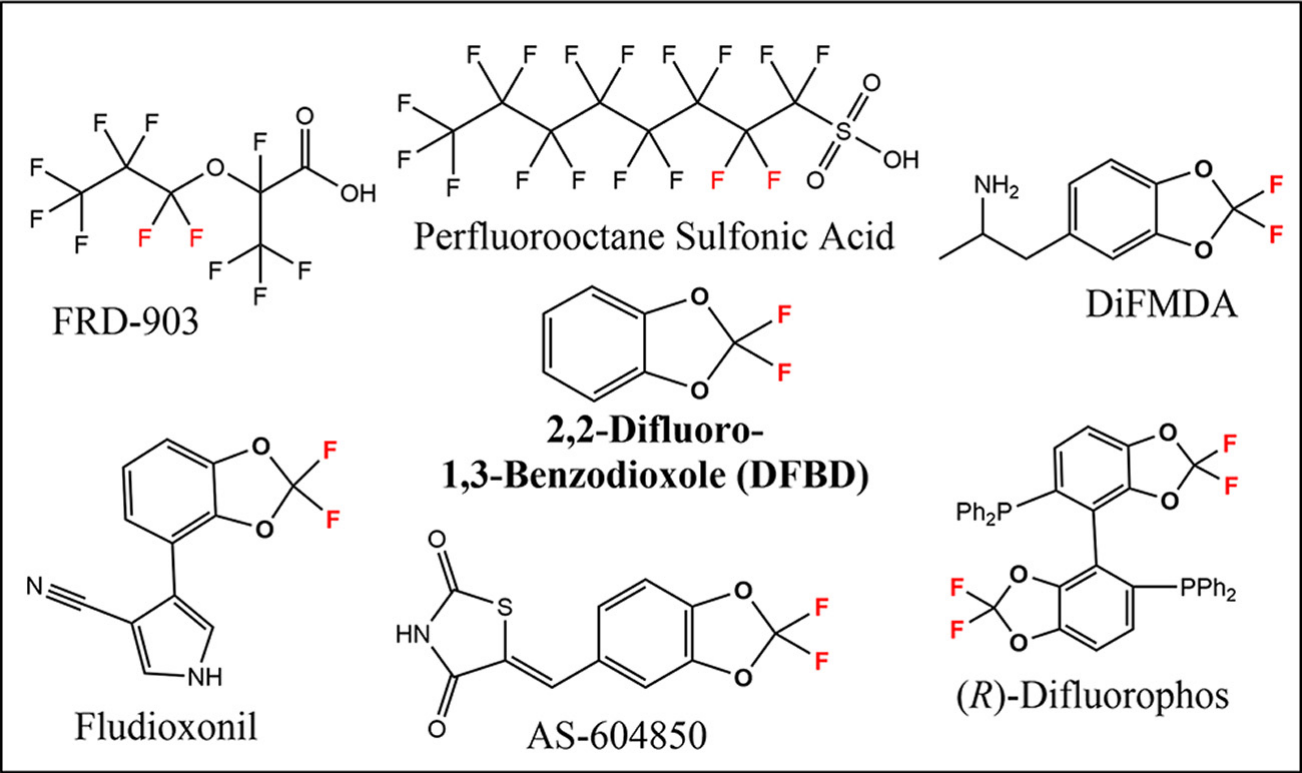
Examples of commercially relevant polyfluorinated compounds. The center compound in bold is 2,2-difluro-1,3-benzodioxole (DFBD). Compounds containing the DFBD moiety shown are the experimental drug DiFMDA (difluoromethylenedioxyamphetamine), the fungicide fludioxonil, the anticancer agent AS-604850, and the reagent for enantioselective synthesis (*R*)-difluorophos.

Difluorinated ethers are common in industrial chemicals, particularly in pharmaceutical and pesticidal compounds. They are of particular interest currently with the introduction of 2,3,3,3-tetrafluoro-2-(heptafluoropropoxy)propanoic acid, commonly known as FRD-903, as a substitute for other perfluorinated alkyl substances (PFAS) such as perfluorooctane sulfonic acid (Fig. 1). A common and emerging class of difluoro ether compounds contains the 2,2-difluoro-1,3-benzodioxole (DFBD) group (9, 10). Commercial DFBD-containing compounds include the fungicide fludioxonil, the anticancer therapeutic AS-604850, the synthetic reagent (*R*)-difluorophos, and the experimental drug difluoromethylenedioxyamphetamine (DiFMDA) (Fig. 1). Many other DFBD-based difluoroethers are in commercial use currently (11). The difluoromethylene group is designed into ring structures because it prolongs the lifetime of the compounds in both the human body and the environment. DFBD-containing pesticides are considered to be moderately recalcitrant, with several studies using enrichments from soil and water showing their disappearance in days to weeks (12, 13). However, those studies did not identify the genes or enzymes responsible for the metabolism.

The defluorination of difluoromethylene carbons in ether linkages is not well studied, and DFBD provides an excellent model for developing understanding. It remains to be established if the fluorines would be displaceable via hydrolytic, reductive, or other mechanisms. The difluorinated carbon atom in DFBD is also bonded to two oxygen atoms. As such, it is completely oxidized and unlikely to undergo oxidative defluorination directly. Defluorination by oxidizing enzymes such as oxygenases is known (14, 15), typically with a carbon atom bonded to a single fluorine atom, unlike in DFBD (16–19). Perfluoroethylene is biodegraded to defluorinated products by soluble methane monooxygenase from Methylosinus trichosporium Ob3B (20). Recently, an elimination mechanism has been proposed to initiate the biodegradation of 3,3,3-trifluoropropionic acid (21), but this mechanism would not apply to the DFBD difluoro-diether group.

For more insights into DFBD defluorination, we chose to examine model Pseudomonas strains for which genomes are available and enzymes have been purified and characterized. The first defluorinating enzyme studied, fluoroacetate defluorinase, was purified and characterized from a Pseudomonas strain (22). In one recent study, fluoroacetate was shown to undergo defluorination by multiple bacterial strains, but difluoro- and trifluoroacetates were uniformly recalcitrant (23). However, Pseudomonas strains are known to contain organochlorine dehalogenases that react with dichloro- and trichloro- substrates (24, 25). Moreover, Pseudomonas genomes are known to encode >25% proteins of unknown function, suggesting novel defluorinating activities may yet be discovered (26, 27). After initially screening several Pseudomonas strains against a small library of fluorinated compounds, we observed significant levels of fluoride anion in the medium with strain Pseudomonas putida F1 and DFBD. This model strain has had its genome sequenced and multiple enzymes characterized in detail (28, 29). The most well-studied enzymes are involved in the metabolism of aromatic hydrocarbons (30), and DFBD contains an aromatic ring. Moreover, the DFBD moiety is known to be quite stable, underlying its incorporation into numerous commercial products (Fig. 1).

The present study focused on DFBD transformation by P. putida F1 and the mechanism by which free fluoride was released into the medium. Using the combined tools of nuclear magnetic resonance (NMR) spectroscopy, gas chromatography (GC), and mass spectrometry (MS), we propose here a novel mechanism underlying fluoride displacement from the DFBD group. It was demonstrated that the only enzyme required for defluorination of DFBD is toluene dioxygenase, and the mechanism elucidated here has, to our knowledge, never been shown previously. Moreover, many environmental bacteria express toluene dioxygenase homologs, suggesting that the mechanism revealed here may be operative with DFBD compounds found in soil and water.

## RESULTS AND DISCUSSION

### Metabolic studies with wild-type Pseudomonas putida F1.

**FIG 2:**
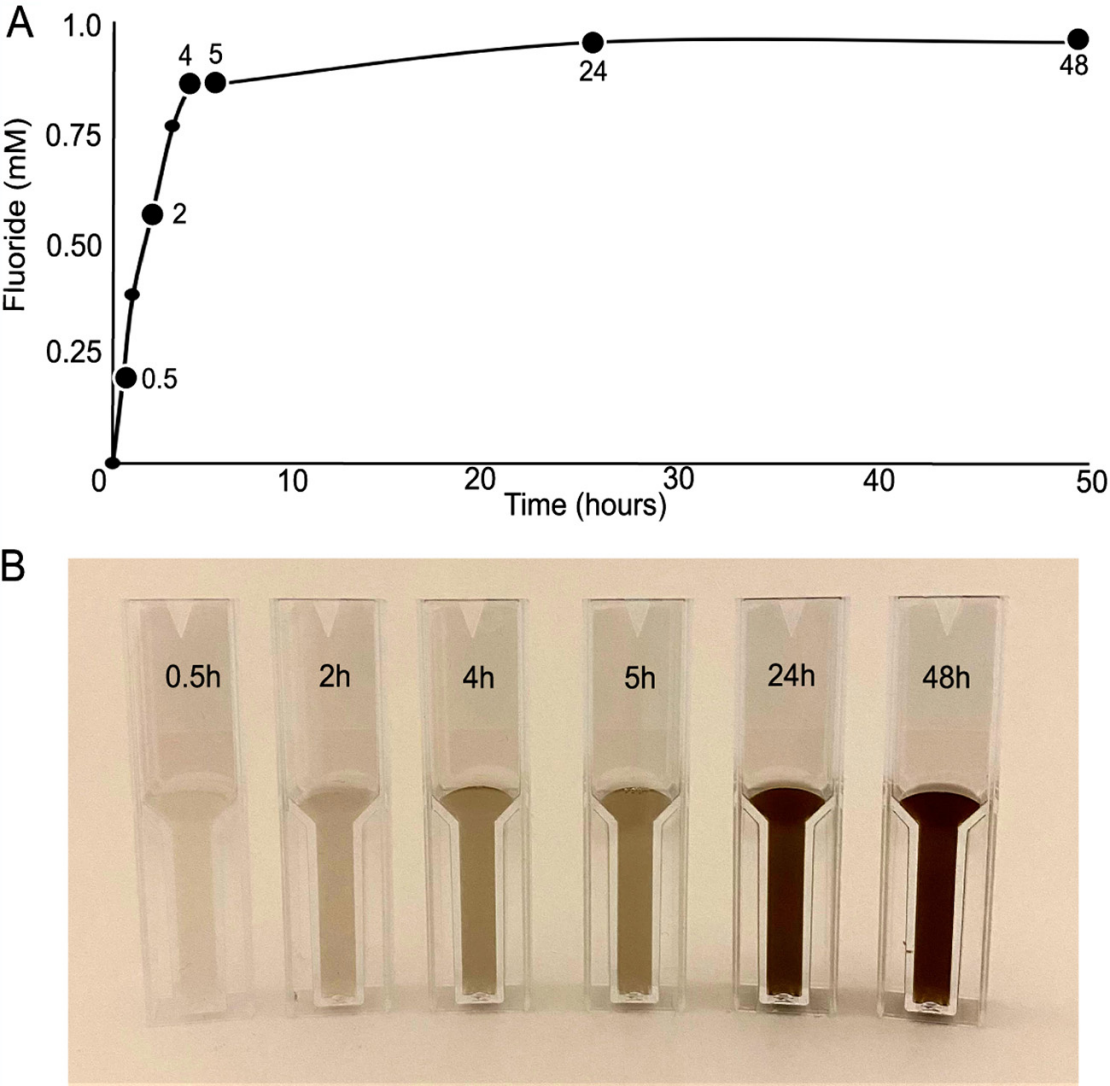
Fluoride release (A) and color change (B) in medium of a culture of Pseudomonas putida F1 incubated with DFBD with shaking over 48 h. P. putida F1 was grown with toluene as the carbon source, which induces the toluene catabolic pathway, and then the culture was switched to DFBD. For fluoride measurements, cells were removed, and the supernatant liquid was analyzed for fluoride, using an ion-specific electrode, as shown in panel A. Aliquots of the culture were taken at the times indicated and stored frozen, and then all were directly photographed, as shown in panel B.

Wild-type cells of P. putida F1 were grown to mid-exponential phase on toluene vapors to induce toluene dioxygenase and related enzymes. The toluene vapor was removed and replaced with 2,2-difluoro-1,3-benzodioxole (DFBD) vapor, and free fluoride ion was detected immediately. Over time, color change of the medium was also observed (see Fig. S1 in the supplemental material). The release of free fluoride ion was measured using a fluoride electrode, which showed a rapid accumulation of fluoride in the first 4 h (Fig. 2A). After this, fluoride in the medium plateaued at ∼1 mM, which was followed by a darkening of the medium (Fig. 2B). Cells grown on l-arginine as a carbon source that were not induced with toluene produced minimal levels of fluoride (0.1 mM over 24 h). Additionally, DFBD was stable in medium in the absence of cells. No fluoride was released, and there was no darkening of medium. When sodium fluoride was added to medium, no color change was observed. In multiple experiments, color change was always linked to fluoride release but lagged behind temporally as shown in Fig. 2.

10.1128/mBio.03001-21.1 FIG S1 Cultures of P. putida F1 grown and induced with toluene and then moved to the respective carbon sources. From left to right, P. putida F1 with vapor bulb containing DFBD, P. putida F1 with vapor bulb containing toluene, and P. putida F1 with no carbon source. Download FIG S1, PDF file, 0.1 MB. Copyright © 2021 Bygd et al. 2021 Bygd et al. https://creativecommons.org/licenses/by/4.0/ This content is distributed under the terms of the Creative Commons Attribution 4.0 International license.

The initial rate of fluoride ion release was 2,100 nmol/h per mg of cell protein. By way of comparison, the rates of initial enzymatic attack on toluene by different Pseudomonas strains are reported to range from 720 to 10,800 nmol/h per mg protein (31). Defluorination of difluoromethylene carbon atoms by microorganisms has rarely been reported, and when it has, it is typically orders of magnitude slower and measured over weeks or months (7, 8). The rapid plateau reached after 4 h suggests that some factor becomes limiting. This argues against a hydrolytic defluorination since that would require only water that would not become limiting. The observations are more consistent with a redox reaction, since NADH or other sources of electrons could conceivably become depleted. The observation that growth on toluene is required suggests a role for one or more components of the toluene metabolic pathway.

### Metabolic studies with mutant P. putida F39/D and Escherichia coli (pDTG601a) expressing toluene dioxygenase.

The observations with wild-type P. putida F1 suggested that one or more components of the toluene degradation pathway were involved in DFBD defluorination and the darkening of the medium. Since the pathway is initiated by toluene dioxygenase (TDO), we first aimed to examine that enzyme’s reaction with DFBD in isolation. Toluene dioxygenase consists of three components that are highly unstable and separate upon cell lysis and purification. They are a flavoprotein reductase (TodA), a ferredoxin (TodB), and the Rieske dioxygenase protein (TodC_1_C_2_) (32). So, an *in vivo* approach was pursued with P. putida F39/D, a derivative of P. putida F1 that lacks a functional second enzyme in the toluene catabolic pathway (33), and the recombinant strain E. coli (pDTG601a), which contains the *todC1C2BA* genes encoding the complete TDO system but no other enzymes of the toluene pathway (29). It is plausible that these two systems could give different results. For example, the TodE catechol oxygenase in P. putida F39/D could hypothetically catalyze ring cleavage leading to defluorination and E. coli DTG601a does not contain that enzyme.

**FIG 3:**
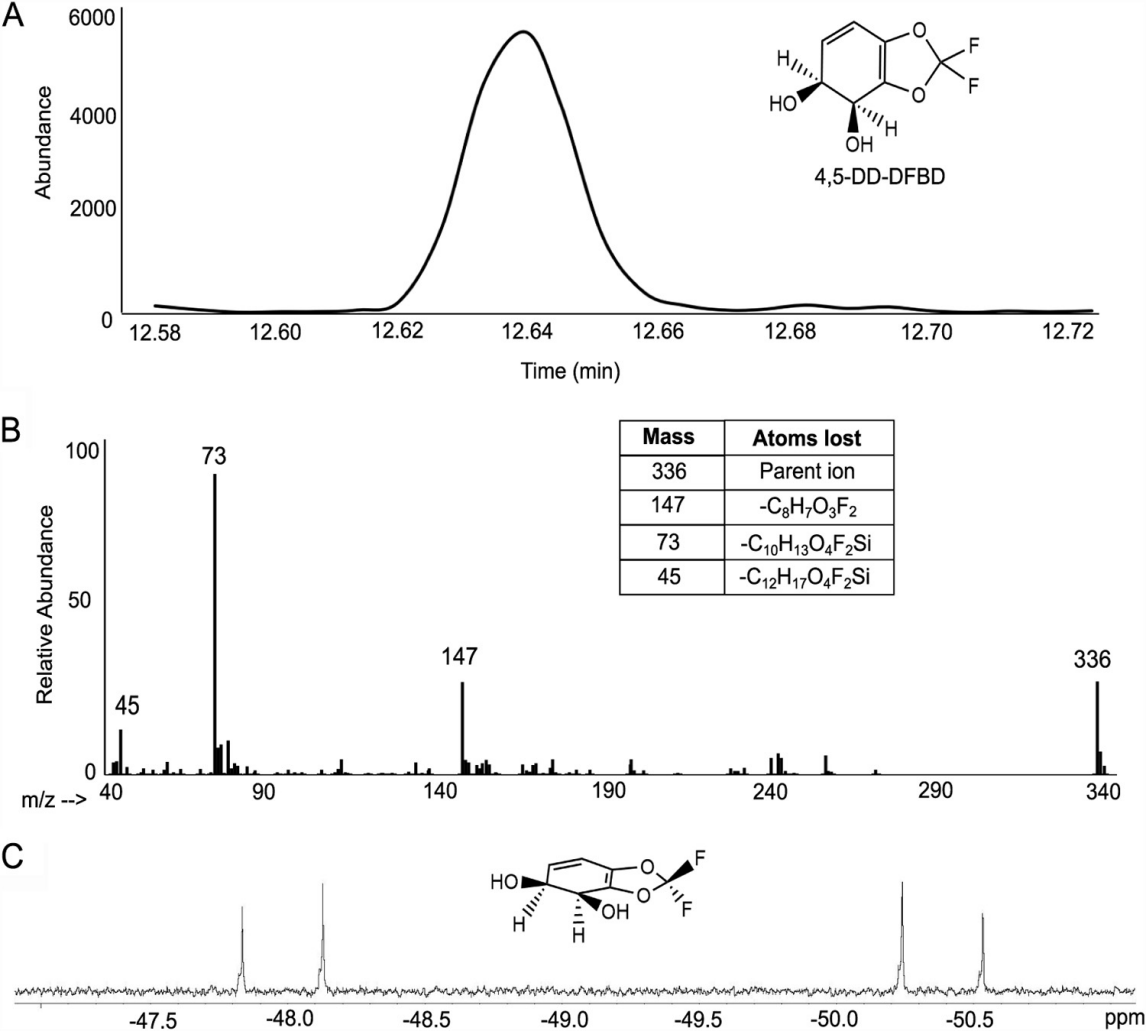
Toluene dioxygenase oxidation of DFBD to a *cis*-dihydrodiol. Analytical demonstration of 4,5-*cis*-dihydroxy-dihydro-2,2-difluoro-1,3-benzodioxole (4,5-DD-DFBD) by GC, MS, and NMR. The structure of the compound is shown in the upper right of panel A. The material analyzed by GC and MS was derivatized as detailed in Materials and Methods. While the data are consistent with a *cis*-dihydrodiol, the absolute stereochemistry has not been determined, and that shown is consistent with dihydrodiols produced by toluene dioxygenase. (A) Gas chromatograph of the extracted product derivatized with trimethylsilane. (B) Mass spectrum of the compound represented by the 12.64-min peak. The parent compound has an *m/z* of 336, and the *m/z* = 337,338 envelope is consistent with 1.1% natural abundance of ^13^C and a compound containing 13 carbon atoms. The disilyl fragment with an *m/z* of 147 is characteristic of compounds with two adjacent derivatized hydroxyl groups. (C) ^19^F-NMR spectrum at 400 MHz of 4,5-DD-DFBD. The two coupled doublets are consistent with two fluorines on differentiated faces, in this case arising from the *cis* configuration of the hydroxyl groups. The 3-dimensional depiction of 4,5-DD-DFBD illustrates the asymmetry imposed by the *cis*-dihydroxylation of DFBD.

P. putida F39/D was grown on l-arginine and induced with toluene vapor before incubation with DFBD. E. coli (pDTG601a) was induced with isopropyl-β-d-thiogalactopyranoside (IPTG) and subsequently incubated with DFBD. In both cases, the media were extracted with ethyl acetate, concentrated using a rotary evaporator, and analyzed by GC-MS and NMR as described in Materials and Methods. The GC-MS and ^19^F-NMR analyses were both consistent with the formation of a 4,5-dihydro-2,2-difluoro-1,3-benzodioxole-4,5-diol (4,5-DD-DFBD) (Fig. 3). Similar results were obtained with the P. putida F39/D and E. coli (pDTG601a) extracts. Because dihydrodiols are subject to decomposition in high temperatures of the GC inlet, extracted medium was derivatized prior to GC-MS analysis. Both the GC retention time and the mass spectrum of the prominent peak at 12.64 min are consistent with its identification as a derivatized dihydrodiol (Fig. 3A and B). Further structural information was gained from ^19^F-NMR that showed that both fluorine atoms were retained in the product (Fig. 3C). The fluorine resonance splitting demonstrated that the plane of the benzene ring was asymmetric, consistent with a *cis*-dihydrodiol. The oxidation of bicyclic fused ring compounds, such as indan (34), by toluene dioxygenase has not been observed to produce bridgehead diols, and the enzyme consistently produces *cis*-dihydrodiols. Therefore, it was predicted that the *cis*-dihydrodiol being formed was most likely a 4,5-*cis*-dihydrodiol.

### Dehydration of the *cis*-dihydrodiol-DFBD.

It was decided that following the fate of 4,5-DD-DFBD would lead to insights into darkening of medium and the defluorination mechanism. Dihydrodiols are known to readily undergo dehydration to yield phenols. The dehydration reaction with substituted benzenes often produces a mixture of two phenols (33). The initial evidence that the DFBD dihydrodiol is unstable came from its low yield and subsequent NMR analysis, initially described in the previous section. When it was dried and taken up in CDCl_3_, a solvent known to be slightly acidic, the ^19^F-NMR spectrum showed a collapse of the fluorine doublet into a single resonance, consistent with a rearomatization of the benzene ring that would occur with dehydration, producing a monohydroxylated aromatic ring (Fig. S6). With rearomatization, the environments of the two fluorine substituents become identical. From the NMR spectrum, it could not be discerned whether DFBD-4-ol, DFBD-5-ol, or both are formed.

10.1128/mBio.03001-21.2 FIG S2 ^1^H-NMR of DFBD-4-ol. Download FIG S2, PDF file, 0.07 MB. Copyright © 2021 Bygd et al. 2021 Bygd et al. https://creativecommons.org/licenses/by/4.0/ This content is distributed under the terms of the Creative Commons Attribution 4.0 International license.

10.1128/mBio.03001-21.3 FIG S3 ^19^F-NMR of DFBD-4-ol. Download FIG S3, PDF file, 0.07 MB. Copyright © 2021 Bygd et al. 2021 Bygd et al. https://creativecommons.org/licenses/by/4.0/ This content is distributed under the terms of the Creative Commons Attribution 4.0 International license.

10.1128/mBio.03001-21.4 FIG S4 ^1^H-NMR of DFBD-5-ol. Download FIG S4, PDF file, 0.08 MB. Copyright © 2021 Bygd et al. 2021 Bygd et al. https://creativecommons.org/licenses/by/4.0/ This content is distributed under the terms of the Creative Commons Attribution 4.0 International license.

10.1128/mBio.03001-21.5 FIG S5 ^19^F-NMR of DFBD-5-ol. Download FIG S5, PDF file, 0.07 MB. Copyright © 2021 Bygd et al. 2021 Bygd et al. https://creativecommons.org/licenses/by/4.0/ This content is distributed under the terms of the Creative Commons Attribution 4.0 International license.

10.1128/mBio.03001-21.6 FIG S6 ^19^F-NMR in CDCl_3_ of the concentrated CD_3_CN solution, previously containing the *cis*-dihydrodiol. Reactions show expected dehydration of the diol in the presence of acid. Download FIG S6, PDF file, 0.1 MB. Copyright © 2021 Bygd et al. 2021 Bygd et al. https://creativecommons.org/licenses/by/4.0/ This content is distributed under the terms of the Creative Commons Attribution 4.0 International license.

**FIG 4:**
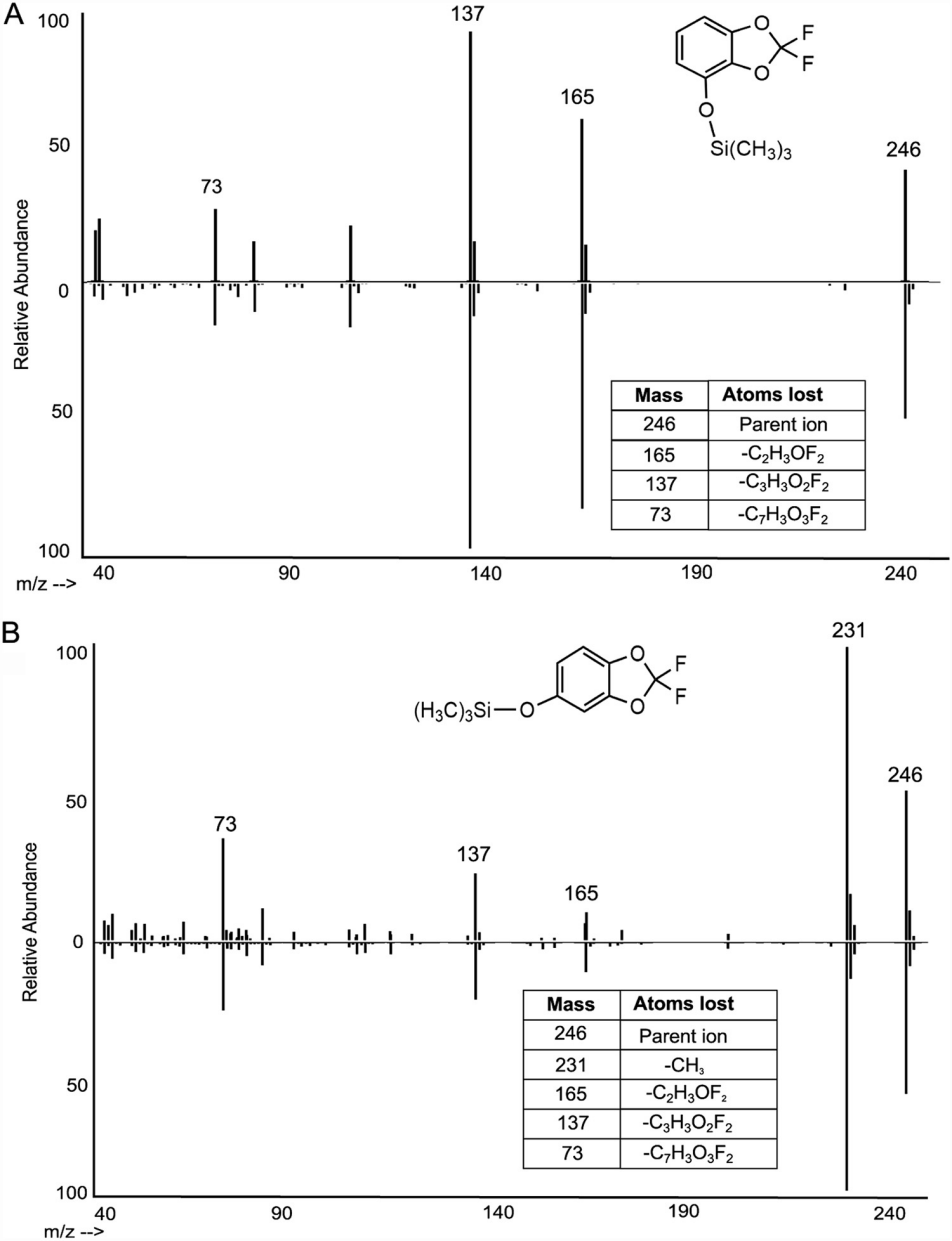
Comparison of dihydrodiol dehydration products from E. coli pDTG601a with synthetic standards following derivatization and mass spectrometry. (A) Mass spectra of trimethylsilane (TMS)-derivatized metabolite from cell cultures incubated with DFBD (top) and standard DFBD-4-ol (bottom). (B) Mass spectra of TMS-derivatized metabolite from cell cultures incubated with DFBD (top) and standard DFBD-5-ol (bottom).

Direct evidence was obtained that both DFBD-4-ol and DFBD-5-ol form in growth medium. This was determined by GC-MS, following derivatization to make both trimethylsilyl derivatives, and in comparison to authentic standards of each (Fig. 4). Together, they are not major products and account for <20% of total products. Moreover, addition of DFBD-4-ol and DFBD-5-ol to P. putida F1 growth medium did not lead to formation of fluoride; both compounds are stable in medium. In addition, no evidence for the metabolism of standard DFBD-4-ol and DFBD-5-ol by P. putida F39/D or E. coli (pDTG601a) in minimal salts basal (MSB) medium was observed. Based on these observations, additional metabolic studies were needed to uncover the mechanism of fluoride displacement.

### E. coli (pDTG602) expressing toluene dioxygenase and diol dehydrogenase.

**FIG 5:**
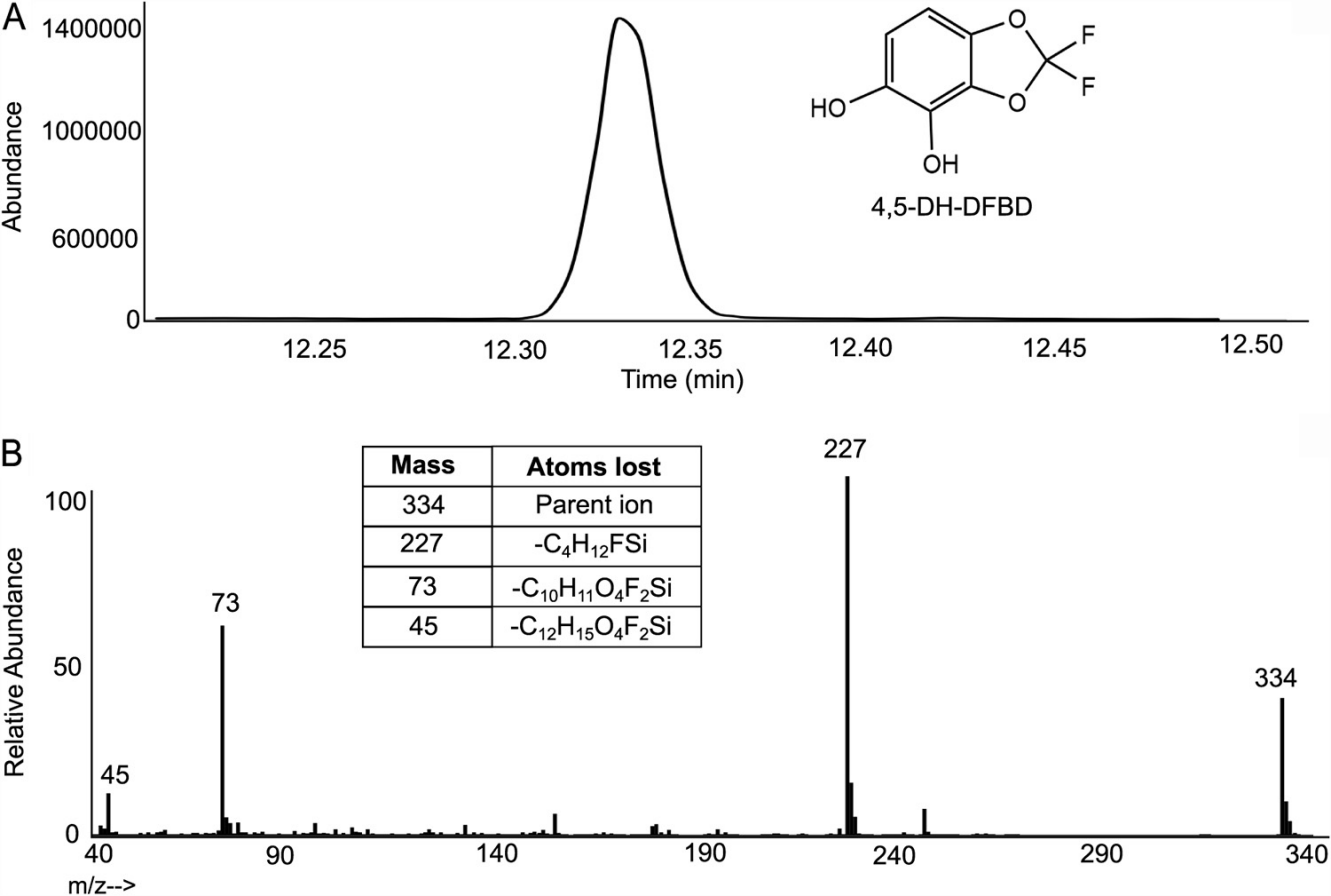
Extracted product derived from E. coli (pDTG602) expressing toluene dioxygenase and toluene dihydrodiol dehydrogenase. (A) Gas chromatogram of the compound identified as TMS-derivatized 4,5-dihydroxy-DFBD. (B) Mass spectrum of TMS-derivatized 4,5-dihydroxy-DFBD. The parent compound shows an *m/z* of 334.

P. putida F1 is known to oxidize *cis*-dihydrodiols to catechols that can oxidize to form products that darken growth medium (29). In that context, we sought to accumulate the catechol from DFBD and determine if that could help explain medium darkening and fluoride release. The experiment used E. coli (pDTG602), which contains genes encoding toluene dioxygenase and dihydrodiol dehydrogenase and is known to accumulate catechols (29). This study produced a metabolite consistent with 4,5-dihydroxy-DFBD (4,5-DH-DFBD), as demonstrated by ^1^H-NMR (Fig. S7), ^19^F-NMR (Fig. S8), and GC-MS after derivatization (Fig. 5). However, this intermediate also maintained the two fluorine substituents on the intact 1,3-dioxolane ring. This was confirmed via GC-MS (Fig. 5) and ^19^F-NMR (Fig. S8). Moreover, similar to the presence of DFBD-4-ol and DFBD-5-ol in E. coli (pDTG601a) cultures, 4,5-dihydroxy-DFBD was formed in only low yield. More importantly, we observed that medium from E. coli pDTG602 incubated with DFBD did darken but not as quickly or to the same extent with as with P. putida F39/D and E. coli (pDTG601a). This suggested that the DFBD-4,5-dihydrodiol is undergoing some other reaction to give a colored product(s) and fluoride.

10.1128/mBio.03001-21.7 FIG S7 ^1^H-NMR of the E. coli pDTG602 supernatant extract containing 4,5-dihydroxy-DFBD and 1,2,3-benzenetriol (pyrogallol). Download FIG S7, PDF file, 0.09 MB. Copyright © 2021 Bygd et al. 2021 Bygd et al. https://creativecommons.org/licenses/by/4.0/ This content is distributed under the terms of the Creative Commons Attribution 4.0 International license.

10.1128/mBio.03001-21.8 FIG S8 ^19^F-NMR of the E. coli pDTG602 supernatant extract containing DFBD-4,5-diol. Fluorine singlet illustrating rearomatized compound with magnetically identical fluorine atoms. Download FIG S8, PDF file, 0.10 MB. Copyright © 2021 Bygd et al. 2021 Bygd et al. https://creativecommons.org/licenses/by/4.0/ This content is distributed under the terms of the Creative Commons Attribution 4.0 International license.

### Identification of pyrogallol, a defluorinated product that explained medium darkening.

**FIG 6:**
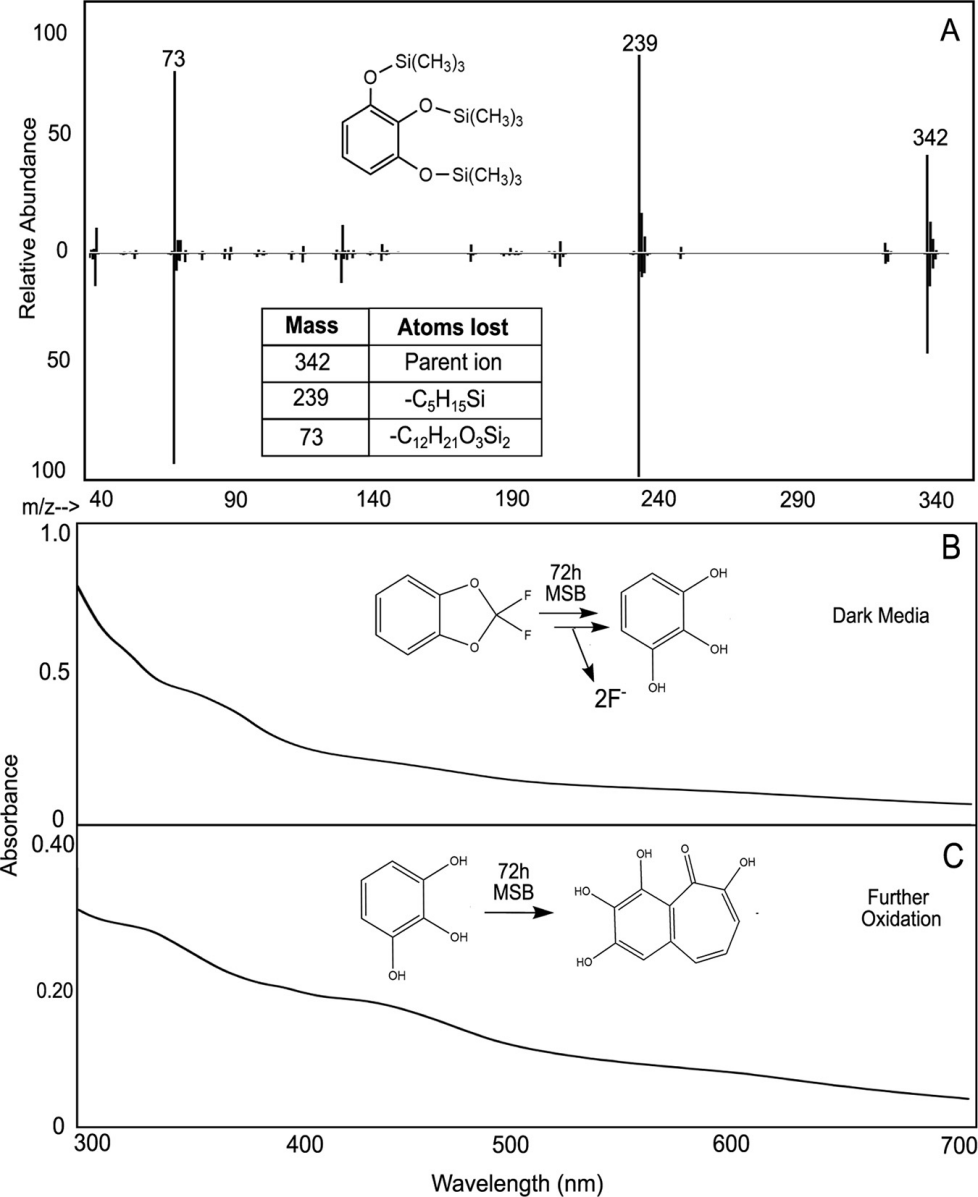
Evidence for the defluorinated aromatic product, pyrogallol, or 1,2,3-benzenetriol. (A) Mass spectrometry fragmentation pattern of derivatized 1,2,3-benzenetriol found in cell cultures from E. coli pDTG601a (top) compared to standard derivatized 1,2,3-benzenetriol (bottom). (B) UV-vis spectrum of E. coli pDTG601a cell culture supernatant after incubation with DFBD for 72 h. (C) UV-vis spectrum of standard 1,2,3-benzenetriol in MSB medium after 72 h. Time course data are shown in Fig. S10.

The volatility of DFBD prevented our determining the stoichiometry of DFBD oxidized to the amount of fluoride released. The initial rate of fluoride release (Fig. 2) was high, and the yields of DFBD-4-ol, DFBD-5-ol, and 4,5-DH-DFBD were low, suggesting that the DFBD-*cis*-4,5-dihydrodiol undergoes another reaction leading to defluorination. In this context, another organic product was sought in cultures of E. coli (pDTG601a) and P. putida F39/D. GC-MS revealed the transient formation of a nonfluorinated product, 1,2,3-benzenetriol, also known as pyrogallol (Fig. 6A). 1,2,3-Benzenetriol is known to undergo spontaneous oxidation in air (35, 36), and our cultures are vigorously shaken to provide a continuous supply of oxygen for the dioxygenase reaction. These oxidation products absorb at varying wavelengths, which leads to dark-colored microbiological medium (37, 38).

10.1128/mBio.03001-21.10 FIG S10 Oxidation products of pyrogallol. (Top panel) ^1^H-NMR of standard purpurogallin. (Bottom panel) UV-vis spectra of auto-oxidation of pyrogallol and purpurogallin in medium. (A) Current knowledge of oxidized species. (B) Time course of pyrogallol (0.1 mM) oxidation in MSB medium over 2.5 h. Absorbance increases uniformly at 320 nm and 440 nm. (C) Time course of purpurogallin (0.02 mM) oxidation in MSB medium over 1.2 h. As the purpurogallin dissolved, an immediate increase was seen at 320 nm (2). Then, the 320 nm decreased and the absorbance at 440 nm increased (3). Download FIG S10, PDF file, 0.2 MB. Copyright © 2021 Bygd et al. 2021 Bygd et al. https://creativecommons.org/licenses/by/4.0/ This content is distributed under the terms of the Creative Commons Attribution 4.0 International license.

To follow pyrogallol formation and decomposition, we used UV-visible (UV-vis) spectroscopy of biological material in comparison to standard pyrogallol and purpurogallin (Fig. 6B and C; see also Fig. S9 and S10). Pyrogallol, prior to oxidation, absorbs only below 300 nm, which is difficult to analyze because DFBD absorbs in the 250- to 300-nm region. So, we analyzed at wavelengths above 300 nm. When added to sterile growth medium, pyrogallol quickly oxidized to a species that absorbs at 320 nm, purportedly purpurogallin (Fig. S10). Over time, this decayed with the appearance of absorbance at 420 to 440 nm, indicating the formation of purpurogallin-quinone (35). Over time, other absorbing species led to the previously described brown to black coloration of pyrogallol medium that was observed here with DFBD in medium in the presence of toluene dioxygenase (Fig. 6B) and standard pyrogallol in medium (Fig. 6C).

10.1128/mBio.03001-21.9 FIG S9 ^1^H-NMR of standard 1,2,3-benzenetriol (pyrogallol). Download FIG S9, PDF file, 0.08 MB. Copyright © 2021 Bygd et al. 2021 Bygd et al. https://creativecommons.org/licenses/by/4.0/ This content is distributed under the terms of the Creative Commons Attribution 4.0 International license.

### Mechanism of defluorination.

A satisfactory explanation for DFBD defluorination had to align several key data. First, fluoride release was immediate and rapid and required toluene-grown cells, and medium darkening lagged temporally. All bacterial strains expressing toluene dioxygenase, even singly, released fluoride and darkened medium. However, known further transformation products of the toluene dioxygenase, which are 4,5-DD-DFBD and the phenols and catechols characterized here, did not undergo defluorination. Indeed, the presence of the enzyme dihydrodiol dehydrogenase, which transformed at least some 4,5-DD-DFBD to the corresponding catechol, diminished fluoride release and color formation. Those observations, together with the immediate fluoride release, indicated that 4,5-DD-DFBD was undergoing another reaction, and that reaction was fast enough to compete with the dehydrogenase kinetically. This previously undescribed reaction pathway had to explain rapid fluoride release, pyrogallol appearance in the medium, and a lag in darkening of the medium.

**FIG 7:**
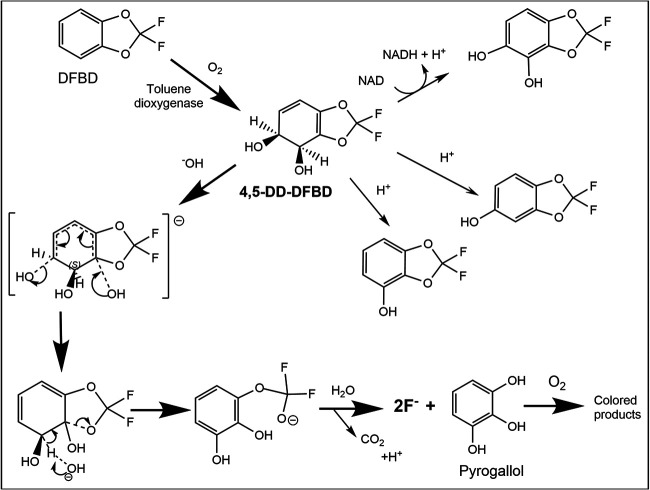
Scheme showing 2,2-difluoro-1,3-benzodioxole (DFBD) oxidation by toluene dioxygenase to 4,5-dihyro-dihydroxy-DFBD (4,5-DD-DFBD) and subsequent reactions producing fluoride ion and dark medium. The major path is highlighted by darker arrows. The three intermediates between 4,5-DD-DFBD and fluoride plus pyrogallol are expected to have a very short lifetime, cannot be demonstrated directly, and represent a proposed mechanism for fluoride and medium darkening. The minor products on the upper right are stable and were demonstrated in this work, as was pyrogallol and the pathway for color formation.

It was not possible to isolate and store 4,5-DD-DFBD due to its extreme instability compared to other bicyclic dihydrodiols (34). Its instability was presumably linked to the highly electron-withdrawing –OCF_2_- moiety adjacent to the dihydrodiol group, precisely the atoms that are lost (Fig. 7). The carbon atom linking the two groups referred to above is a bridgehead carbon. This bridgehead carbon would be highly susceptible to nucleophilic attack by a hydroxide anion in solution. The resultant trihydroxy compound (middle left, Fig. 7) is expected to decompose with C-O breakage, rearomatization, and opening of the five-membered 1,3-dioxole ring. We propose here that these are spontaneous reactions, driven by the electrophilicity of the bridgehead carbon atom and the stabilization energy inherent in rearomatization that occurs with C-O bond cleavage. However, we cannot rule out the possibility of a cell component assisting in hydroxide attack on the bridgehead carbon. Once the C-O bond cleaves, the resultant –OCF_2_O- moiety is highly unstable, to undergo very rapid hydrolysis to produce carbon dioxide and the two fluorine atoms as fluoride. Pyrogallol, the other product, is not colored but over time will oxidize to a range of colored products that darken the medium (Fig. 7).

### Potential for DFBD defluorination by other aromatic oxygenase-containing bacteria.

A question naturally arises as to whether DFBD defluorination by this mechanism is common, since genes encoding the oxygenation of toluene and other aromatic hydrocarbons are widespread in bacterial genomes (39–41). In addition to dioxygenation, many bacteria oxidize toluene via monooxygenases (39). Given the stability of monohydroxy and catecholic DFBD metabolites, we did not expect strains with monooxygenases to defluorinate DFBD. To test this hypothesis, Burkholderia cepacia G4, containing toluene-2-monoxygenase, was grown on glucose and induced with toluene. After 24 h of incubation with DFBD, this strain did not release fluoride or create a dark color in the medium.

Dioxygenases that oxidize multiring substrates were considered to be better candidates to test for DFBD defluorination. Defluorination and darkening of the medium were not observed with Pseudomonas sp. strain NCIB 9816 or Pseudomonas sp. strain 9816-11, each of which expresses a naphthalene dioxygenase. Biphenyl dioxygenases that have different amino acid sequences were also tested with two bacteria, Burkholderia xenovorans LB400 and Sphingobium yanoikuyae B1. After induction with biphenyl and incubation with DFBD for 24 h, no fluoride release or color change was observed with either strain.

### Identifying enzymes with greater similarity to the toluene dioxygenase from P. putida F1.

Toluene dioxygenase is known to be a Rieske oxygenase, a very common class of proteins in aerobic bacteria (39–41). The Rieske oxygenases are composed of two protein subunits. The cofactors and the active site are within the larger, or alpha-, subunit. The alpha-subunit contains a Rieske iron sulfur cluster and a mononuclear iron center. Since the alpha-subunit of Rieske dioxygenases determines substrate specificity (42, 43), sequence comparisons with this subunit are most germane to the question as to which enzymes and organisms will defluorinate DFBD.

The amino acid sequence of the P. putida F1 toluene dioxygenase alpha-subunit (WP_012052601.1) was used to search the Pseudomonas genome database (22, 23) for homologs to the alpha-subunit of toluene dioxygenase on 6 August 2021 using DIAMOND-BLAST. The closer the sequence to the toluene dioxygenase studied here, the more likelihood that the active site would bind and oxygenate DFBD in a manner leading to defluorination (Table 1). This search identified proteins with amino acid sequences that were 100% identical in the following Pseudomonas strains with the locus tags given in the parentheses: Pseudomonas monteilii SB3101 (X970_10585), *P. monteilii* SB3078 (X969_10930), P. putida YKD221 (TR32_RS03575), Pseudomonas sp. strain NBRC 111125 (APH29_RS14695), and P. putida UV4/95 (CBP06_RS10555). Published substrate specificity studies with P. putida UV4/95 have shown the same substrate spectrum as P. putida F1 (44), consistent with the 100% sequence identity of the dioxygenase alpha-subunits.

**TABLE 1:** Homologs of the toluene dioxygenase alpha-subunit (WP_012052601.1) with high sequence identity over the majority of the sequence length^*a*^

Percent identity	No. of sequences	Query sequence coverage
100%	6	100%
>90%	9	100%
>80%	19	>97%
>70%	51	>96%
>60%	177	>96%
>50%	293	>95%

a The sequences searched were compiled from three sources: Pseudomonas genome database, RHObase database, and NCBI nonredundant protein database. Percent identity was determined by pairwise BLAST (49), and the query sequence coverage refers to the extent over which the two sequences aligned.

Next, we queried the RHObase, or Ring-Hydroxylating Oxygenase database, which focuses specifically on characterized Rieske oxygenases using the term “toluene dioxygenase” on 7 August 2021. This identified several enzymes denoted as toluene dioxygenase or nitrotoluene dioxygenases. One of these, from Pseudomonas putida DOT-T1E (ADI95397), was 100% identical to the alpha-subunit of toluene dioxygenase from P. putida F1.

The large GenBank nonredundant database, with 419,490,021 sequences as of 6 August 2021, was searched to determine the approximate number of homologs and particularly close sequence matches. A BLAST search yielded over 1,200 homologous sequences with an E value lower than e^−50^, indicating they matched extremely well. Of those, 293 showed sequence identity of >50% and 9 showed sequence identity of >90% (Table 1). Closely related sequences are found in Pseudomonas, *Comamonas*, *Sphingomonas*, *Paraburkholderia*, *Nocardioides*, and *Rhodococcus*.

Next, the Rieske dioxygenases in the bacteria that were tested here and found not to release fluoride were analyzed for amino acid sequence relatedness to the P. putida F1 toluene dioxygenase. The biphenyl dioxygenase from Sphingobium yanoikuyae B1 is 37% identical in amino acid sequence to toluene dioxygenase via pairwise BLAST alignment. The Pseudomonas sp. strain NCIB 9816 naphthalene dioxygenase is 32% identical to the toluene dioxygenase alpha-subunit. The Paraburkholderia xenovorans LB400 biphenyl dioxygenase is 65% identical.

It is not currently known how much a sequence might deviate from 100% identity, and if slightly different, what specific requirements would ensure reactivity with DFBD at the 4,5-position leading to defluorination. Further experimental work is warranted to test other bacteria with closely related Rieske dioxygenases. It is possible that docking and molecular dynamics simulations could also be done to predict enzyme reactivity and regioselectivity with DFBD (45, 46), but those detailed computational experiments are beyond the scope of the present study. A search of the Protein Data Bank for homologs of toluene dioxygenase yielded four distinct Rieske dioxygenases with X-ray structures and >64% sequence identity. Based on the bioinformatic data presented here and the wide diversity of bacteria known to express Rieske dioxygenases, we suggest that the DFBD moiety in natural environments might be subject to the type of metabolism revealed here with P. putida F1.

### Conclusions.

Pseudomonas strains and oxygenases have the potential to catalyze defluorination reactions in unexpected ways as demonstrated here. Previously, Pseudomonas sp. strain T-12 expressing toluene dioxygenase was shown to hydroxylate a carbon bearing a fluorine substituent, leading to defluorination by *gem* elimination (17). In this study, a difluorinated ether was shown to be defluorinated by P. putida F1 via a different mechanism but also demonstrated to be dependent upon toluene dioxygenase. Bioinformatic analyses strongly suggest that DFBD and its derivatives will undergo defluorination by dioxygenases found in other known Pseudomonas strains and in natural environments.

## MATERIALS AND METHODS

### Bacterial strains, growth, and manipulations.

Bacteria, growth substrates, enzymes expressed, and references are compiled in Table 2. Further specific details on growth and manipulations are provided below. Pseudomonas putida F1 was grown on Luria-Bertani (LB) plates and then grown overnight on minimal salts basal medium (MSB) (47) with carbon supplied via a vapor bulb containing toluene, as previously described (48). This 10-ml culture was then added to an additional 25 ml of MSB, and the toluene bulb was replaced with a 2,2-difluoro-1,3-benzodioxole bulb. This flask was incubated at 30°C with shaking at 200 rpm. Aliquots of the culture were taken out at various time points. Samples intended for measuring fluoride release were centrifuged to remove cells, and the supernatant was removed and placed into a clean tube. For photographs of color change, the medium aliquots were taken directly and then stored at −20°C to be photographed simultaneously. Pseudomonas putida F39/D was originally streaked onto an LB plate and grown at 28°C. For liquid cultures, this strain was grown on MSB and 0.2% (wt/vol) l-arginine. A saturated 10-ml culture of P. putida F39/D, grown overnight, was added to 200 ml of MSB and 0.2% (wt/vol) arginine and allowed to grow to log phase at 30°C. Once log phase was attained, the culture was induced with toluene for 1 h. After induction, the toluene bulb was replaced with a DFBD bulb. After 2 h of incubation with DFBD, the cells were pelleted, and the supernatant was removed.

**TABLE 2:** Bacterial strains used, growth conditions selected, and known enzymes expressed under the conditions specified

Bacterial strain	Growth substrate/inducer	Relevant enzyme(s) expressed^*a*^	Reference(s)
Pseudomonas putida F1	l-Arginine	Toluene metabolism enzymes at very low levels	52
Pseudomonas putida F1	Toluene	Toluene pathway and regulatory proteins	52
Pseudomonas putida F39/D	Toluene	All toluene pathway enzymes with mutation in the diol dehydrogenase (TodD)	48
Escherichia coli K-12^*b*^	d-Glucose	NA^*d*^	
Escherichia coli JM109 (pDTG601a)	IPTG^*c*^	Toluene dioxygenase	29
Escherichia coli JM109 (pDTG602)	IPTG^*c*^	Toluene dioxygenase and diol dehydrogenase	29
Pseudomonas sp. NCIB 9816	Naphthalene	Naphthalene dioxygenase pathway	53, 54
Pseudomonas sp. NCIB 9816-11	Naphthalene	Naphthalene enzymes with diol dehydrogenase mutation	53, 54
Burkholderia cepacia G4	Toluene	Toluene-2-monoxygenase pathway	55
Sphingobium yanoikuyae B1	Biphenyl	Biphenyl dioxygenase pathway	56
Paraburkholderia xenovorans LB400	Biphenyl	Biphenyl dioxygenase pathway	57

a Based on peer-reviewed references.

b No aromatic ring oxygenases known to us induced with E. coli grown on glucose.

c IPTG, isopropyl-β-*d*-1-thiogalactopyranoside; added to induce expression of the specified enzyme(s) indicated.

d NA, not applicable.

E. coli pDTG601a was maintained on LB plates with 100 μg/ml ampicillin at 37°C. For experimental use, it was grown overnight on LB with ampicillin liquid, cells were pelleted, and then the cells were resuspended in MSB, 0.2% (wt/vol) glucose, and 100 μg/ml ampicillin. After growing at 37°C for 2 h, this strain was induced with 1 mM IPTG for 1 h. After induction, a DFBD vapor bulb was added to the flask and the culture was left to incubate for a further 2 h. E. coli pDTG602 cells were maintained on LB-ampicillin plates at 37°C. For experimental use, this strain was grown overnight on MSB medium with glucose and 100 μg/ml ampicillin. This overnight culture was used to inoculate a culture of 50 ml of MSB, glucose, and ampicillin and induced with IPTG for 1 h. After induction, DFBD was added in vapor form. Aliquots were taken at various time points and left to incubate overnight. Pseudomonas sp. NCIB 9816 and 9816-11 were maintained on LB plates at 28°C. NCIB 9816 was grown to log phase on 100 ml of MSB and naphthalene for induction of naphthalene dioxygenase. Subsequently, the culture was filtered through glass wool to remove naphthalene crystals and a vapor bulb of DFBD was added to the culture. NCIB 9816-11 was grown on MSB medium and 15 mM pyruvate. This culture was added to 25 ml of MSB and induced with 0.2% naphthalene. This culture was also filtered through glass wool before adding the DFBD vapor bulb, and then the culture was left to incubate for 24 h. Burkholderia cepacia G4 was maintained on LB plates at 28°C. It was grown in MSB and 0.2% glucose overnight. This culture was added to 25 ml of MSB and induced with toluene for 1 h. Sphingobium yanoikuyae B1 was maintained on LB plates at 28°C. Overnight, this strain was grown on MSB and biphenyl, which induces the biphenyl dioxygenase. Paraburkholderia xenovorans LB400 was maintained on nutrient agar plates at 28°C. Overnight, this strain was grown on MSB and biphenyl, which induces the biphenyl dioxygenase enzyme.

### Chemicals.

2,2-Difluoro-1,3-benzodioxole (DFBD) was purchased from Matrix Scientific and ^1^H-NMR indicated 97% purity. 2,2-Difluoro-1,3-benzodioxole-4-ol and 2,2-difluoro-1,3-benzodioxole-5-ol were from AstaTech with 95% purity. 1,2,3-Benzenetriol was purchased from Mallinckrodt with a purity of ∼95%. Purpurogallin was from Cayman Chemical and had a purity of 95%, as determined by ^1^H-NMR. *N*,*O*-Bis(trimethylsilyl)trifluoroacetamide was purchased from Fluka Analytical and had a purity of 99%. CDCl_3_ and CD_3_CN were obtained from Cambridge Isotope Laboratories, Inc., and had >99% purity. Methyl-*tert*-butyl ether (MTBE) and ethyl acetate (EtOH), solvents used for GC-MS, were from Sigma-Aldrich, both high-performance liquid chromatography (HPLC) grade, >99% pure. Toluene used for specified cell growth and induction was from Fisher Chemical and is 99% pure. Sodium fluoride (NaF) used for fluoride standards was obtained from Aldrich Chemical company, 99% pure. d-Glucose, used for growth of E. coli recombinant strains, was purchased from Sigma-Aldrich and was 99.5% pure. IPTG was obtained from Gold Bio. l-Arginine, used for growth of Pseudomonas strains, is from Calbiochem. Sodium pyruvate is from Alfa-Aesar with a purity of 99%. Biphenyl was from Spectrum Chemical and had a purity of 99%. Naphthalene was from Alfa-Aesar with a purity of 99%.

### Nuclear magnetic resonance (NMR) experiments.

Cell culture supernatants from both E. coli pDTG601a and P. putida F39/D after incubation with DFBD were extracted with an equal volume of ethyl acetate (EtOAc). In each case, the aqueous layer was drawn off and the organic layer was dried (MgSO_4_), transferred to a round-bottom flask, and rotary evaporated. The residue was taken into CD_3_CN for initial ^1^H- and ^19^F-NMR acquisitions on a Varian INOVA 500-MHz NMR spectrometer. The initial ^1^H-NMR spectra were too complex to identify signals due to 4,5-dihydro-DFBD-4,5-diol, but ^19^F-NMR (376 MHz, Fig. 3C; see also Fig. S6 in the supplemental material for CD_3_CN) shows a clean AB pattern: 46.65 ppm (d, *J* = 110.8 Hz) and −49.05 (d, *J *= 110.8 Hz) for the CF_2_ fluorine atoms.

Cell culture supernatants from E. coli pDTG602 were extracted with MTBE, dried with anhydrous MgSO_4_, and then rotary evaporated. Subsequently, the CD_3_CN solutions were concentrated and then directly dissolved in CDCl_3_. The products were as follows: ^1^H-NMR (400 MHz, CDCl_3_), 1,2,3-benzenetriol; 6.68 ppm (t, *J* = 8.0 Hz), 6.48 ppm (d, *J* = 8.2 Hz), 5.24 ppm (bs), 5.17 ppm (bs), and DFBD-ols, 6.57 ppm (d, *J* = 8.4 Hz), 6.51 ppm (d, *J* = 8.4 Hz), and buried OH broad singlets, ^19^F-NMR (400 MHz, CDCl_3_), −50.31 ppm (s), CF_2_. In the proton NMR, the high field doublet at 6.51 ppm is partly buried in a neighboring peak. Additional significant peaks that do not describe the 4,5-DD-DFBD are shown to be 1,2,3-benzenetriol (Fig. S7 and S9).

### GC-MS experiments.

For gas chromatography and mass spectrometry (GC-MS), cell culture supernatants were extracted with methyl-*tert*-butyl ether (MTBE). Equal volumes of organic and aqueous phases were used. Extraction was done directly in glass GC vials, which were shaken vigorously, and then the organic layer was drawn off and placed in a clean vial. One microliter of derivatizing agent, *N*,*O*-bis(trimethylsilyl)trifluoroacetamide, was added to each extracted sample. Product ion spectra were identified in positive ion mode on an HP6890 gas chromatograph with an HP5973 MS detector. GC conditions were as follows: helium gas, 1 ml/min; HP-1ms column (100% dimethylpolysiloxane capillary; 30 m by 250 μm by 0.25 μm); starting temperature 50°C with a ramp of 10°C/min to 320°C. Standards run on the GC-MS used the same program specified above. 2,2-Difluoro-1,3-benzodioxole-5-ol and 2,2-difluoro-1,3-benzodioxole-4-ol were prepared by placing a small volume of each into separate GC vials with 500 μl of MTBE. One microliter of the derivatizing agent was added to each vial. Pyrogallol (1,2,3-benzenetriol) was prepared similarly, by putting a small solid amount of the compound into 500 μl of MTBE and adding 1 μl of derivatizing agent.

### Fluoride measurements.

Fluoride ion measurements were determined using an ISE Ionplus Sure-Flow Orion fluoride probe from Thermo Fisher and the Orion Star A214 meter. To perform fluoride measurements, 1 ml of cell culture was pelleted. The supernatant was removed and placed into a 5-ml tube with 1 ml of TISAB (58.5 g/liter NaCl, 15 g/liter CH_3_COOH, 66 g/liter CH_3_COONa, and 1 g/liter 1,2-cyclohexane diaminetetraacetic acid). Calibrations of the probe were made with standard concentrations ranging from 10^−5^ M to 10^−2^ M. Two standards were required for calibration. Standards were made with sodium fluoride in MSB medium.

### UV-vis spectroscopy.

UV-vis absorption scans were performed on a Cary 3500 double-beam UV-vis spectrophotometer containing a Xenon flash lamp from Agilent. Measurements of supernatant from an E. coli pDTG601a culture were taken after 72 h of incubation with DFBD. An aliquot was taken from the cell culture and pelleted to remove cells. The supernatant was removed and placed in a 1.5-ml disposable cuvette. The sample was then measured (300 to 700 nm) in increments of 5 nm. Standard 1,2,3-benzenetriol was prepared to a concentration of 0.1 M in water. To scan this compound, 1 ml of MSB was placed in a 1.5-ml disposable cuvette and 1,2,3-benzenetriol stock solution was added to a final concentration of 0.1 mM. This cuvette of MSB and 0.1 mM pyrogallol was left to sit at room temperature for 72 h and measured after that.

### Bioinformatic methods.

BLAST was used to query the NCBI GenBank nonredundant database (49). DIAMOND-BLAST was used to query the Pseudomonas genome database (50). The RHObase, or Ring-Hydroxylating Oxygenase database, was queried with a word search (51). All hits obtained from the searches were compiled manually. Comparisons with toluene dioxygenase and other single enzymes were carried out using pairwise BLAST (49) on the National Center for Biotechnology Information server.
