# Supplementary material for: Unexpected Mechanism of Biodegradation and Defluorination of 2,2-Difluoro-1,3-Benzodioxole by Pseudomonas putida F1
Source: mBio. 2021 Nov 16;12(6):e03001-21. doi: 10.1128/mBio.03001-21 (PMC8593668; doi:10.1128/mBio.03001-21)
Supplement: FIG S1 [file mbio.03001-21-sf001.pdf]

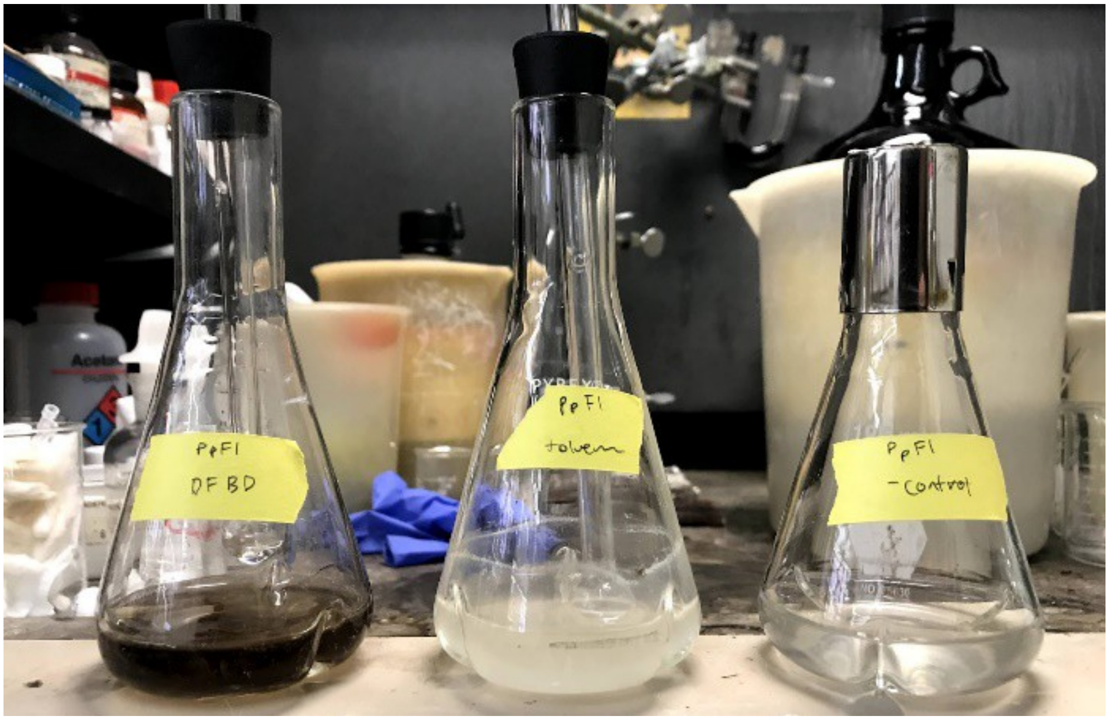

Figure S1. Cultures of *P. putida* F1 grown and induced with toluene, then moved to the respective carbon sources. From left to right: *P. putida* F1 with vapor bulb containing DFBD, *P. putida* F1 with vapor bulb containing toluene, *P. putida* F1 with no carbon source.
