# Supplementary figures and images for: Unexpected Mechanism of Biodegradation and Defluorination of 2,2-Difluoro-1,3-Benzodioxole by Pseudomonas putida F1
Source: mBio. 2021 Nov 16;12(6):e03001-21. doi: 10.1128/mBio.03001-21 (PMC8593668; doi:10.1128/mBio.03001-21)

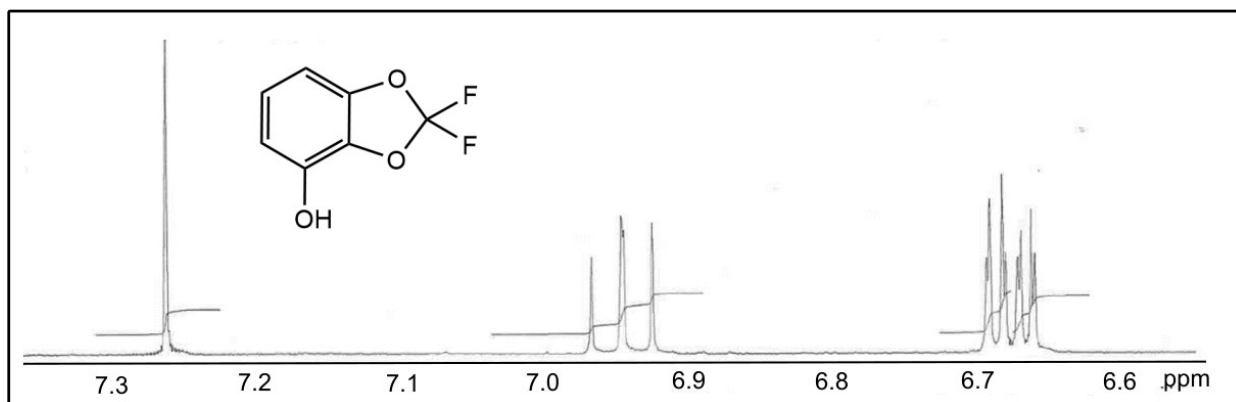

**Figure S2.**  $^1\text{H}$ -NMR in  $\text{CDCl}_3$  of the DFBD-4-ol standard

Supplement: FIG S2 [file mbio.03001-21-sf002.pdf]

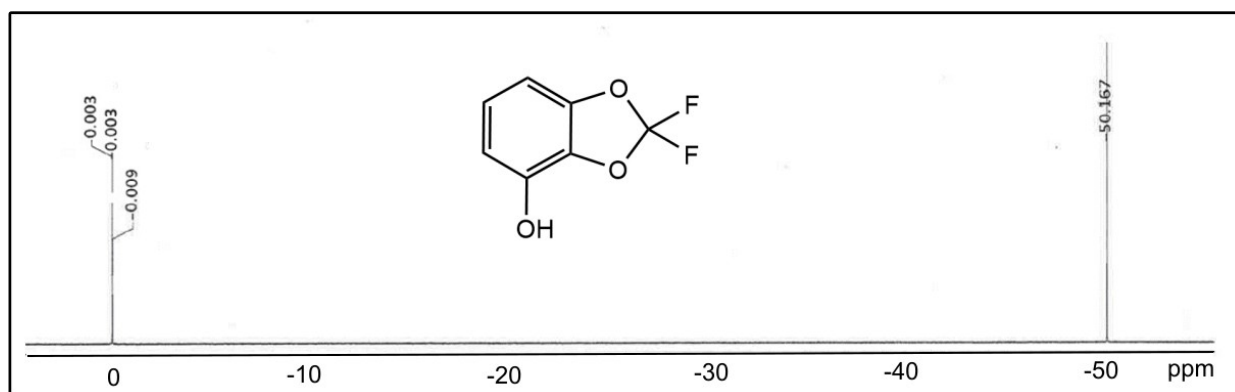

**Figure 3S.**  $^{19}\text{F}$ -NMR of the DFBD-4-ol standard

Supplement: FIG S3 [file mbio.03001-21-sf003.pdf]

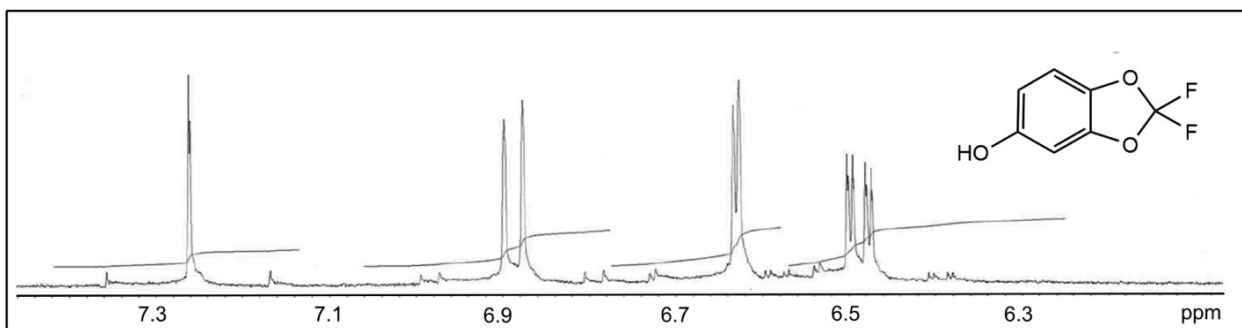

**Figure 4S.**  $^1\text{H}$ -NMR of the DFBD-5-ol standard

Supplement: FIG S4 [file mbio.03001-21-sf004.pdf]

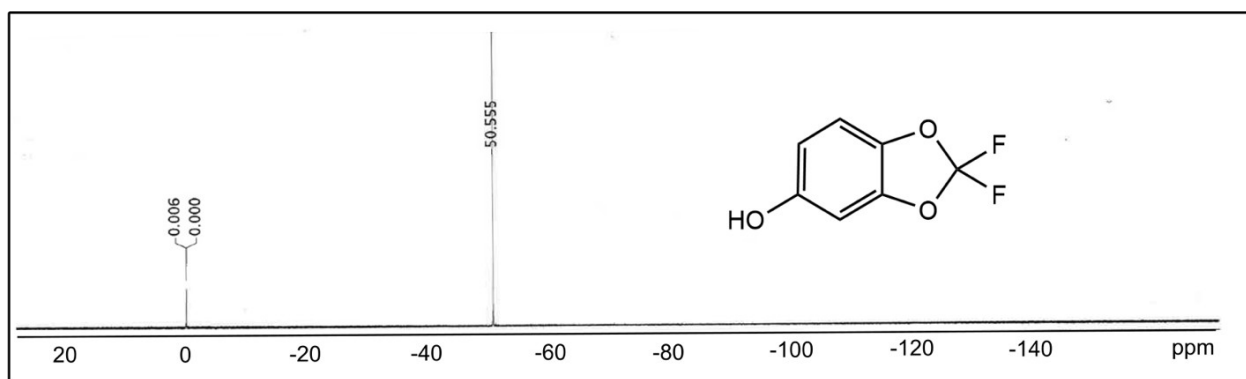

**Figure S5.**  $^{19}\text{F}$ -NMR of the DFBD-5-ol standard.

Supplement: FIG S5 [file mbio.03001-21-sf005.pdf]

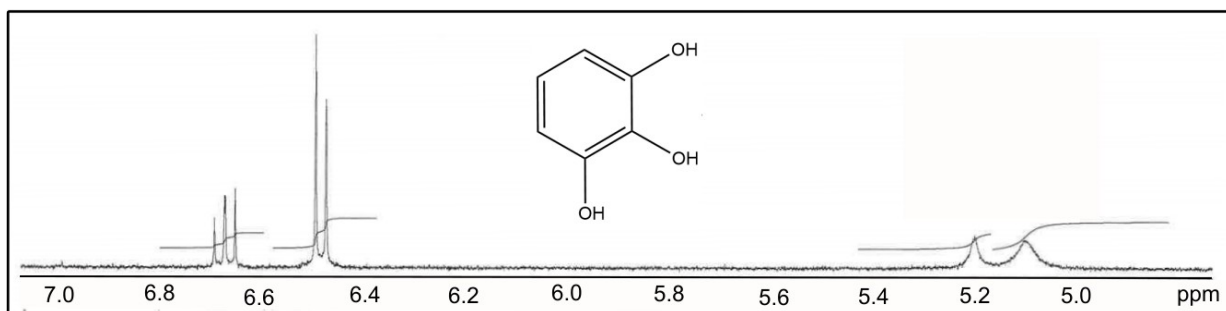

**Figure S9.**  $^1\text{H}$ -NMR of standard 1,2,3-benzenetriol (pyrogallol).

Supplement: FIG S9 [file mbio.03001-21-sf009.pdf]
