# Supplementary material for: Unexpected Mechanism of Biodegradation and Defluorination of 2,2-Difluoro-1,3-Benzodioxole by Pseudomonas putida F1
Source: mBio. 2021 Nov 16;12(6):e03001-21. doi: 10.1128/mBio.03001-21 (PMC8593668; doi:10.1128/mBio.03001-21)
Supplement: FIG S6 [file mbio.03001-21-sf006.pdf]

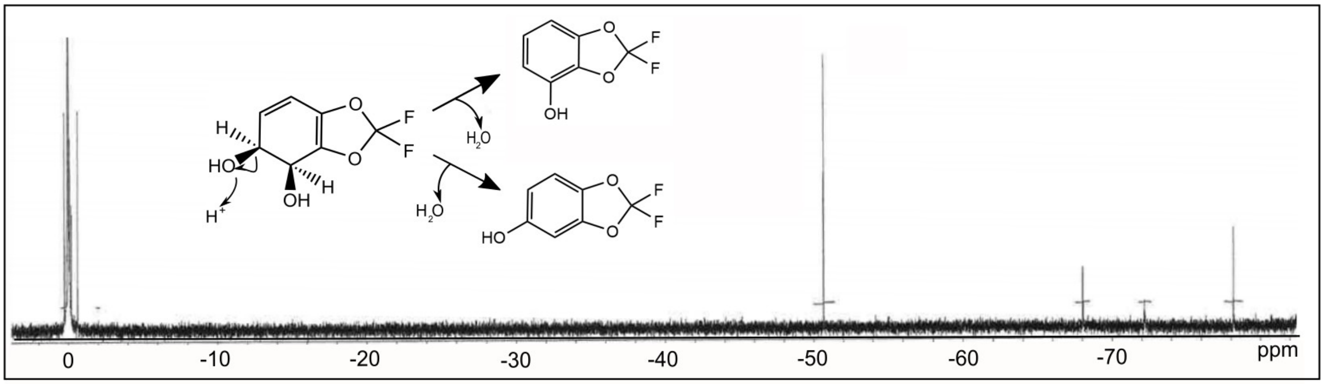

Figure S6. ^{19}F-NMR in $CDCl_3$ of the concentrated CD_3CN solution, previously containing the *cis*-dihydrodiol. Reactions show expected dehydration of the diol in the presence of acid
