# Supplementary material for: Unexpected Mechanism of Biodegradation and Defluorination of 2,2-Difluoro-1,3-Benzodioxole by Pseudomonas putida F1
Source: mBio. 2021 Nov 16;12(6):e03001-21. doi: 10.1128/mBio.03001-21 (PMC8593668; doi:10.1128/mBio.03001-21)
Supplement: FIG S7 [file mbio.03001-21-sf007.pdf]

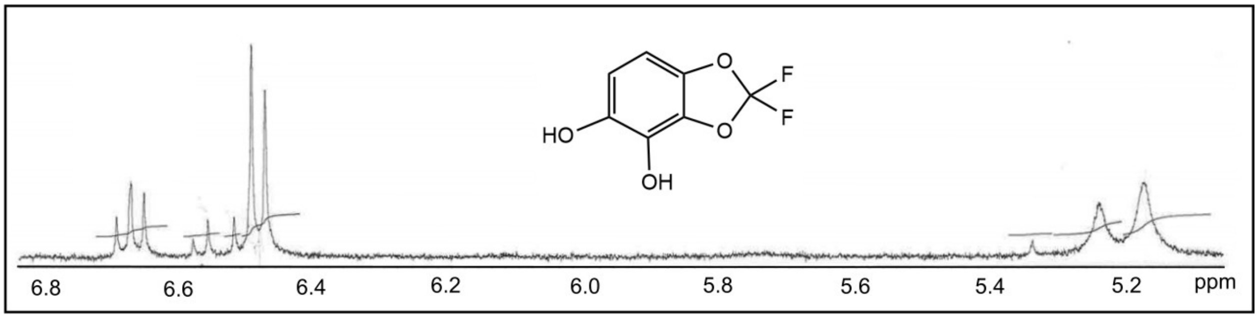

Figure S7. ^{1}H-NMR of the *E. coli* pDTG602 supernatant extract containing 4,5-dihydroxy-DFBD and 1,2,3-benzenetriol (pyrogallol).
