# Supplementary material for: Unexpected Mechanism of Biodegradation and Defluorination of 2,2-Difluoro-1,3-Benzodioxole by Pseudomonas putida F1
Source: mBio. 2021 Nov 16;12(6):e03001-21. doi: 10.1128/mBio.03001-21 (PMC8593668; doi:10.1128/mBio.03001-21)
Supplement: FIG S8 [file mbio.03001-21-sf008.pdf]

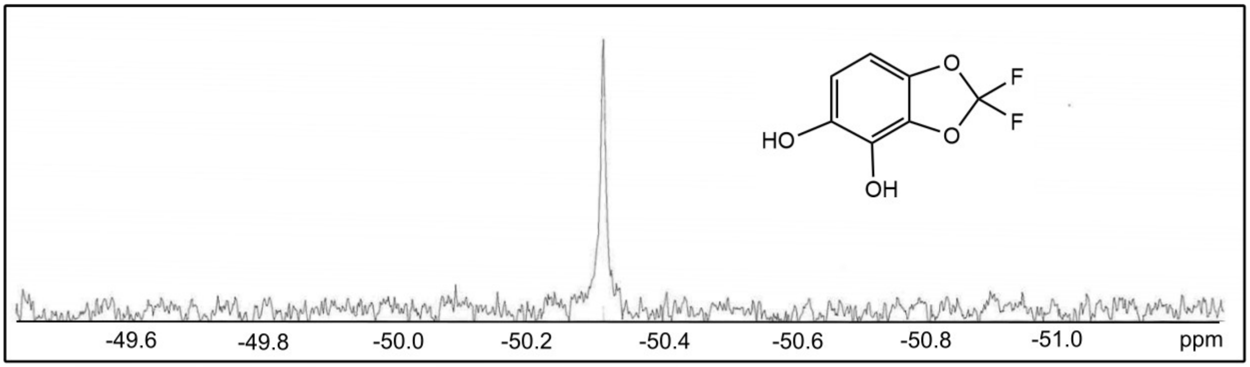

Figure 8S. ^{19}F-NMR of the the *E. coli* pDTG602 supernatant extract containing DFBD-4,5-diol. Fluorine singlet illustrating rearomatized compound with magnetically identical fluroineatoms.
