# Supplementary material for: Unexpected Mechanism of Biodegradation and Defluorination of 2,2-Difluoro-1,3-Benzodioxole by Pseudomonas putida F1
Source: mBio. 2021 Nov 16;12(6):e03001-21. doi: 10.1128/mBio.03001-21 (PMC8593668; doi:10.1128/mBio.03001-21)
Supplement: FIG S10 [file mbio.03001-21-sf010.pdf]

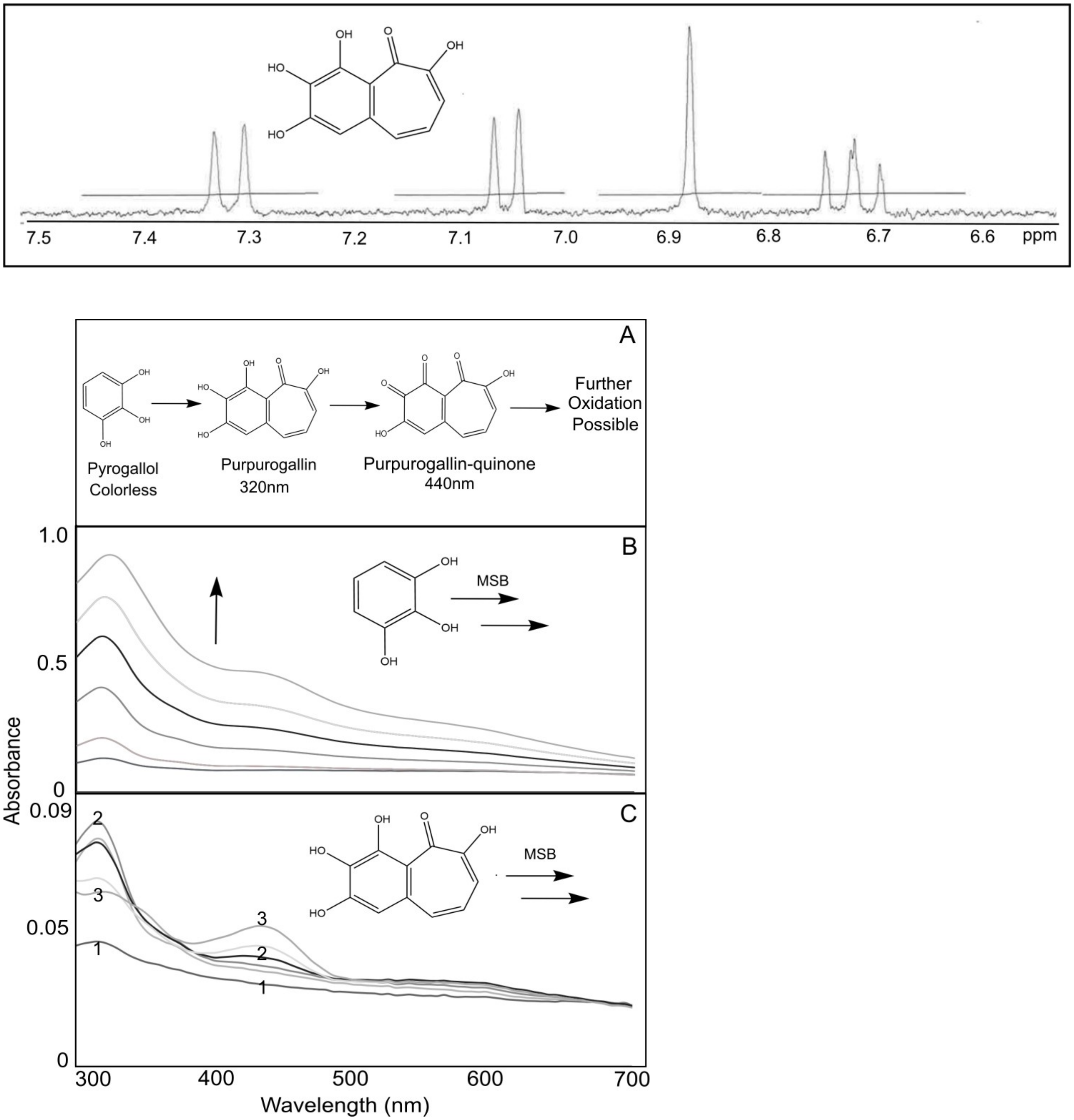

Figure S10. Oxidation products of pyrogallol. Top panel, ^{1}H-NMR of standard purpurogallin. Bottom panel, UV-vis spectra of auto-oxidation of pyrogallol and pupruogallin in media. (A) Current knowledge of oxidized species. (B) Time course of pyrogallol (0.1 mM) oxidation in MSB media over 2.5 hours. Absorbance increases uniformly at 320 nm and 440 nm. (C) Time course of purpurogallin (0.02 mM) oxidation in MSB media over 1.2 hours. As the purpurogallin dissolved,an immediate increase was seen at 320 nm (2). Then, the 320 nm decreased and the absorbance at440 nm increased (3).
